# Whole Process of Standardization of Diffusion-Weighted Imaging: Phantom Validation and Clinical Application According to the QIBA Profile

**DOI:** 10.3390/diagnostics14060583

**Published:** 2024-03-09

**Authors:** Se Jin Choi, Kyung Won Kim, Yousun Ko, Young Chul Cho, Ji Sung Jang, Hyemin Ahn, Dong Wook Kim, Mi Young Kim

**Affiliations:** 1 Department of Radiology and Research Institute of Radiology, Asan Medical Center, University of Ulsan College of Medicine, Seoul 05505, Republic of Korea; sejin711@gmail.com (S.J.C.); jsjang@amc.seoul.kr (J.S.J.); ypc99093@naver.com (H.A.); fsnoruen@gmail.com (D.W.K.); 2 Biomedical Research Center, Asan Institute for Life Sciences, Asan Medical Center, University of Ulsan College of Medicine, Seoul 05505, Republic of Korea; cjsakura@naver.com; 3 Department of Radiology, Asan Medical Institute of Convergence Science and Technology (AMIST), Asan Medical Center, University of Ulsan College of Medicine, Seoul 05505, Republic of Korea; gornrnrn@gmail.com

**Keywords:** apparent diffusion coefficient, diffusion-weighted imaging, magnetic resonance imaging, phantom, Quantitative Imaging Biomarker Alliance

## Abstract

**Background:** To use the apparent diffusion coefficient (ADC) as reliable biomarkers, validation of MRI equipment performance and clinical acquisition protocols should be performed prior to application in patients. This study aims to validate various MRI equipment and clinical brain protocols for diffusion weighted imaging (DWI) using commercial phantom, and confirm the validated protocols in patients’ images. **Methods:** The performance of four different scanners and clinical brain protocols were validated using a Quantitative Imaging Biomarker Alliance (QIBA) diffusion phantom and cloud-based analysis tool. We evaluated the performance metrics regarding accuracy and repeatability of ADC measurement using QIBA profile. The validated clinical brain protocols were applied to 17 patients, and image quality and repeatability of ADC were assessed. **Results:** The MRI equipment performance of all four MRI scanners demonstrated high accuracy in ADC measurement (ADC bias, −2.3% to −0.4%), excellent linear correlation to the reference ADC value (slope, 0.9 to 1.0; R^2^, 0.999–1.000), and high short-term repeatability [within-subject-coefficient-of-variation (wCV), 0% to 0.3%]. The clinical protocols were also validated by fulfilling QIBA claims with high accuracy (ADC bias, −3.1% to −0.7%) and robust repeatability (wCV, 0% to 0.1%). Brain DWI acquired using the validated clinical protocols showed ideal image quality (mean score ≥ 2.9) and good repeatability (wCV, 1.8–2.2). **Conclusions:** The whole process of standardization of DWI demonstrated the robustness of ADC with high accuracy and repeatability across diverse MRI equipment and clinical protocols in accordance with the QIBA claims.

## 1. Introduction

Diffusion-weighted imaging (DWI) is a functional sequence in magnetic resonance imaging (MRI) that reflects the diffusivity of the water molecule which is affected by tissue cellularity, tissue microstructure, fluid viscosity, cellular membrane permeability, and blood flow [1,2,3,4]. The apparent diffusion coefficient (ADC) value is the functional parameter calculated by mean diffusivity along three orthogonal directions from DWI. In current clinical practice, DWI is primarily used for lesion detection and characterization. However, recent advances in therapeutics have increased the demand to use the ADC as a quantitative imaging biomarker for treatment response assessment in an objective manner considering that ADC value reflecting the cellularity of lesion [2,5,6,7]. For example, chemotherapy-related changes in tumors begins with alteration in tissue cellularity changes followed by lesion size changes [1,6,8]. Treatment response assessment of tumors requires longitudinal monitoring with quantitative biomarkers at multiple time points [7,9]. Therefore, imaging biomarkers for treatment response assessment should be validated for both accuracy (i.e., how close the ADCs are to the true values) and precision (i.e., how close the ADCs are between repeatable measurements).

DWI are relatively susceptible to MRI scanners’ specific features such as SNR or the calibration of high strength diffusion gradient system that affects the cross-term factors, as well as sequence parameters [10,11,12,13]. The ADC serves as a reflection of the diffusivity of a lesion, and is calculated by applying the signal intensity measured in DWI to an exponential equation. Consequently, when the signal intensity in DWI is subject to variation due to numerous technical factors, the reliability and repeatability of ADC measurements become challenging to ensure. Thus, it is important to maintain constant MRI scanners and sequence parameters to use DWI and ADC as biomarkers [14,15]. In response to these needs, the Quantitative Imaging Biomarker Alliance (QIBA) issued the QIBA DWI profile for the standardized imaging protocols for DWI acquisition [16]. The DWI acquisition protocols for phantom suggested by the QIBA (hereafter referred to as QIBA phantom protocol) are designed to minimize difference of ADC values regardless of the type of MRI scanner and software version. The validation of MRI equipment performance and clinical protocols is performed using a phantom. The QIBA developed the QIBA diffusion phantom to validate the accuracy and repeatability of DWI acquisition and ADC measurement [9,17,18]. Along with phantom development, a cloud-based tool was also developed for standardized phantom image analysis. After fulfillment of the MRI scanner, clinical brain protocols scanned with validated MRI equipment can be validated for patient scanning in an image quality assurance (QA) process. The final step of QA is to review patient DWI for image quality and ADC measurement repeatability. 

The QIBA phantom protocol primarily focuses on the validation of MRI scanners rather than its applicability in clinical practice. On the contrary, clinical acquisition protocols for brain DWI employed in each institution for routine clinical practice (hereafter referred to as clinical brain protocols) differ from the QIBA phantom protocol. The clinical brain protocols prioritize practicality in real-world settings, and further variability is introduced across institutions due to the diverse MRI vendors and software. Thus, the accuracy and precision of ADC measurements should be validated with these clinical brain protocols to use ADC as imaging biomarker.

To date, there is sparse evidence of the use of the QIBA diffusion phantom and standardized phantom image analysis tools in real-world practice and clinical trial settings [16,18,19]. A few previous studies have concentrated on the repeatability and reproducibility of ADC values and the validation of MRI equipment using the QIBA phantom. However, to utilize ADC values as an imaging biomarker, it is essential to the standardization process of the validation of DWI and to confirm the feasibility of clinical imaging protocols.

Therefore, we performed this study using the QIBA diffusion phantom to validate four MRI scanners from different vendors with the following goals: (1) validation of MRI equipment performance using the QIBA phantom protocol with a phantom, (2) validation of clinical brain protocol in regard to the accuracy and repeatability with a phantom, and (3) application of the validated clinical brain protocols for patient scanning and image QA. 

## 2. Materials and Methods

### 2.1. Overall Study Scheme

**Figure 1:**
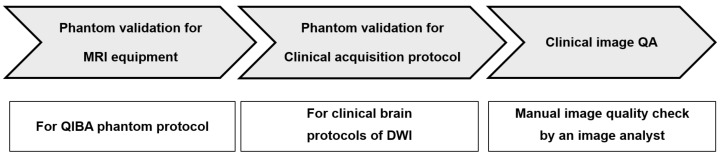
Whole process of image standardization of DWI/ADC measurement using phantom validation and clinical application.

This study was composed of three steps, as illustrated in Figure 1. First, we evaluated the performance of various MRI scanners according to the QIBA claims of the QIBA profile using the QIBA phantom. Second, the clinical brain protocols for DWI using various MRI scanners were validated according to the QIBA claims of the QIBA profile using the QIBA phantom. Finally, we applied the validated clinical brain protocols to patient scanning in a clinical trial and performed image QA for the acquired DWI scans. 

### 2.2. Phantom Preparation

In this study, we used a commercially available phantom (CaliberMRI, Inc., Boulder, CO, USA) developed by the National Institute of Standards and Technology (NIST) with support and input from the National Cancer Institute and QIBA (hereafter referred as QIBA diffusion phantom) [16,19]. The detailed information about the phantom and preparation method for temperature stability are presented in Supplementary Material S1.

### 2.3. DWI Acquisition Using the QIBA Phantom

Table 1 summarizes the detailed information of image acquisition parameters for the QIBA phantom protocol. When scanning the phantom using the QIBA phantom protocol, all acquisition parameters kept the QIBA phantom protocol except that number of excitations (NEX) were 1 rather than 2 in 3.0-T scanner. The QIBA phantom protocol specified TE as shortest, and all scans were obtained with TE < 60 ms in this study. 

Table 2 presented information of the MRI systems and protocols for brain DWI suggested by QIBA and variable MRI vendors. A total of three 3-T platforms (SIGNA Architect, GE Healthcare; Ingenia, Philips Healthcare; Magnetom Vida, Siemens Medical Solutions) and one 1.5-T platform (Magnetom Avanto, Siemens Medical Solutions) were used for DWI. 

DWI phantom images were acquired using two acquisition protocols: (1) the QIBA phantom protocol for MRI equipment performance evaluation (Table 1) and (2) the clinical brain protocols for evaluation of DWI acquisition and ADC measurement for routine practice (Table 2). According to the QIBA profile, short-term (intra-exam) and long-term (multiday) repeatability were evaluated using QIBA phantom protocol. We conducted four examinations for each scanner using the QIBA phantom on the same day and repeated the exams in the same manner one month later for each protocol. The specifications of the clinical brain protocols are consistent with the QIBA profile claims and recommendations for brain DWI. Clinical brain protocol met the ideal or target specification of QIBA brain profile, except acceptable fulfillment for gap thickness (2 mm), acquired matrix (128 × 128), and number of average (1). 

### 2.4. Quantitative Analysis of Phantom Data 

#### 2.4.1. Phantom Data Processing

For standardized analysis of quantitative DWI phantom data, we requested the analysis of DWI data to the commercially available cloud-based quality assessment software (CaliberMRI, Inc., qCal-MR quality control (QC) Software, Boulder, CO, USA www.qmri.com, accessed on 12 August 2021) [20]. The data and figures were adopted from the quality assessment report with permission from CaliberMRI. The analytical method of the quality assessment software is summarized in Supplementary Material S2.

#### 2.4.2. Phantom Data Analysis 

Phantom data analysis was performed for both the QIBA phantom protocol for MRI equipment performance evaluation and the DWI clinical brain protocols for routine clinical practice. All phantom data analyses followed the QIBA profile using the QIBA claims. For phantom data acquired using the QIBA phantom protocol, protocol compliance was checked for whether the acquired MRI met the recommended acquisition parameters of the QIBA phantom protocol. A radiologist (S.J.C) performed visual inspection to assess the image quality of all phantom DWI data on site for the presence of all required DWI series, number of b-values as suggested by QIBA (Table 2), and presence of artifacts to interfere evaluation of performance metrics by quality assessment software. 

The definition and equation of the quantitative DWI performance metrics are presented in Table 3 [9,16]. The quantitative DWI performance metrics included the bias in ADC measurement (ADC bias), the measurement repeatability estimated by the repeatability coefficient (RC) and the within-subject coefficient-of-variation (wCV), linearity, b-value dependence, random measurement error, and signal-to-noise ratio (SNR). 

ADC bias is an estimate of measurement error that is calculated by the difference between the average measurement and its true value. For efficient data demonstration, bias divided by the true value is presented as a percentage (%bias). Repeatability is the representative of measurement precision in a repeated exam on the same or a similar examination condition over a short period of time with the same measurement procedure [9,16]. In a phantom study, short-term repeatability within the examination were assessed by calculating the RC and wCV. Linearity refers to the ability to provide measured quantity values that are directly proportional to the value of the reference standard. Precision reflects the closeness of agreement between the measured values obtained by replicate measurements on the same or similar experimental units under specified conditions. In this study, we assessed random error as an indicator of precision. Evaluation of the SNR provides a relative system performance metric and confirms that the MRI equipment is adequate to measure ADC bias without incremental bias due to a low SNR [16]. SNR was calculated as spatial mean of signal image divided by mean of noise image. 

### 2.5. Application of Validated Clinical Brain Protocols to Patients 

This prospective study was approved by the institutional review board of our hospital (2017-0467), and informed consent was obtained from all eligible patients. Patients who visited the institution for evaluation of cerebral infarction and agreed to participate in the clinical study were included. After the clinical study trial period, we retrospectively collected brain DWI scans from these included patients. The scans were conducted using the clinical brain protocol for the initial evaluation and follow-up of cerebral/cerebellar infarction between March 2013 and September 2020 were included for analysis. The mean age of the human subjects was 66.1 years (range, 38–87 years), and male subjects accounted for 58.8% (10/17). A total of 55 DWI scans were performed in these 17 patients, comprising 20 scans from 3.0-T machines and 35 scans from a 1.5-T machine. Details about the brain DWI scan protocol are presented in Table 2. If there were any image degradation caused by patients’ motion, susceptibility or eddy current distortion, immediate corrections were made on-site and the exams were retaken [21]. 

We evaluated the image quality and then calculated the RC and wCV of each image. DWI data quality was evaluated by an experience radiologist (K.W.K.) for the items presented by the QIBA using a 3-point scale (1, Unacceptable; 2, Acceptable; 3, Ideal) [14]. Details of these items are summarized in the Supplementary Material S3.

**Figure 2:**
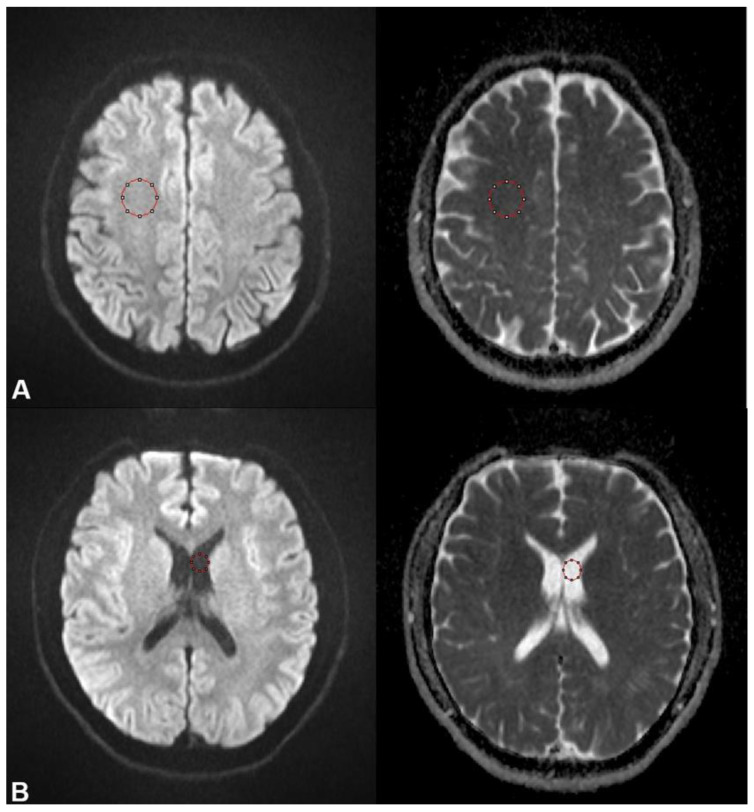
Example of ROI segmentation for brain white matter and CSF. A circled ROI was drawn at frontoparietal white matter (**A**) and lateral ventricle (**B**) on a b-1000 image and then transferred to an ADC map. The mean and standard deviation were calculated with the software.

After evaluating the quality of the DWI data, ADC values were extracted using AsanJ-Stroke Software (Asan Image Metrics, Seoul, Korea; assessed at https://datasharing.aim-aicro.com/strokevolumetry, accessed on 31 May 2023) [22], which was developed based on ImageJ (NIH, Bethesda, MD, USA). In each subject, a radiologist drew two ROIs in the non-diseased healthy brain hemisphere—a circle with a 20 mm diameter at the center of the frontoparietal white matter and another with a 10 mm diameter in the cerebrospinal fluid (CSF) of the lateral ventricle—for ADC measurements. The ROIs were manually drawn on the b = 1000 s/mm^2^ image considering lesion location (Figure 2). The ROIs were saved in separate files and then subsequently applied to the equivalent site of the ADC map. AsanJ provides the ADC pixel histogram as well as the ADC mean and standard deviation using data from the ADC map. Using extracted data from the ADC, performance metrics according to the QIBA profile were evaluated. To evaluate the long-term repeatability of each scanner, the RC and wCV were calculated as the QIBA profile. And we compared the measured ADC value with reference ADC value of white matter and CSF [23,24]. 

## 3. Results

### 3.1. Validation of MRI Equipment Performance

#### 3.1.1. Protocol Compliance and Image Quality

**Figure 3:**
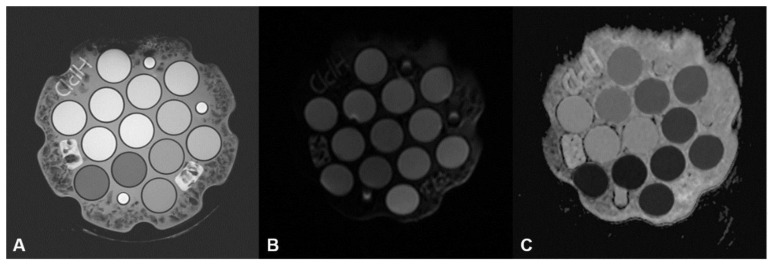
Representative images of DWI and ADC maps of phantom. T2-weighted images (**A**) and DWI with b-value of 0, 500, 1000 (**B**), 1500, 2000 s/mm^2^ were obtained according to QIBA profile. Exponential ADC maps (**C**) were generated using all b-values and a mono-exponential model.

Phantom DWI data from the four MRI systems were successfully acquired satisfying the QIBA profile without artifact. Quality assessment software evaluated the protocol compliance according to the QIBA profile before calculation of performance parameters. All phantom DWI data acquired from the four MRI scanners using the QIBA phantom protocol passed the protocol compliance. No parameters exceeded the limits of the QIBA claims (Supplementary Figure S1). For qualitative inspection, we identified four DWI series and confirmed that each was composed of five b-values. DWI data from the four vendors had appropriate image quality without significant artifacts. Representative images of DWI and ADC maps are shown in Figure 3.

#### 3.1.2. Key Quantitative DWI Performance Metrics

The ADC VOI statistics reports and derived graph of DWI data generated by the quality assessment company are attached as Supplementary Figure S1. The analysis report consists of ADC statistics and representative graphs of ADC values based on axial position, varying concentration of solution, and repeatability parameter of ADC. The performance metrics results of all scanners are summarized in Table 4. All four MRI scanners met the conformance of the QIBA DWI claims. 

The ADC bias of the four MRI scanners ranged from −2.3% to −0.4%, which were within the QIBA DWI claims (<absolute value of 3.6). The short-term repeatability of all four MRI scanners with wCV ranging from 0% to 0.3% also met the QIBA claims (wCV ≤ 0.5%). There was an excellent linear correlation between the measured ADC values and the true values (i.e., NIST values), with slope ranging from 0.98–1.0 and R^2^ ranging from 0.999–1.000 in all MRI scanners, which met the QIBA claim (R^2^ > 0.9 and slope 0.9–1.0). The max b-value dependence, ranging from 0.1% to 1.5%, was within the QIBA claim limit (≤2%). The random measurement errors ranging from 0.5% to 1.7% also met the QIBA claim (≤2%). The SNR of all four MRI scanners were high (56.0–230.1), which met the QIBA claim (≥45). 

### 3.2. Validation of Clinical Brain Protocols

#### 3.2.1. Protocol Compliance and Image Quality

For evaluation of the clinical brain protocols, we have verified the protocol compliance manually, because the QC software was optimized for the QIBA protocol. For qualitative inspection, we identified four DWI series and confirmed that each series was composed of two b-values, b = 0 s/mm^2^ and b = 1000 s/mm^2^. DWI data from the four vendors had appropriate image quality without significant artifacts.

#### 3.2.2. Key Quantitative DWI Performance Metrics

The ADC VOI statistics reports derived from the qCal-MR QC software are presented in Supplementary Figure S2. As presented in Table 5, the results of the performance metrics of all clinical brain protocols are as follows: ADC bias values (ADC bias, −3.1% to −0.7%), short-term repeatability (wCV, 0–0.1%), linearity (R^2^, 0.995–0.998; slope, 0.95–1.00), random measurement error (0.4–0.8%), and SNR (145.6–380.3). These performance metrics met the QIBA claims. However, the max b-value dependence could not be calculated for the clinical brain protocols because there were only two b-values. 

### 3.3. Quality Assurance for Acquired Patient DWI

**Figure 4:**
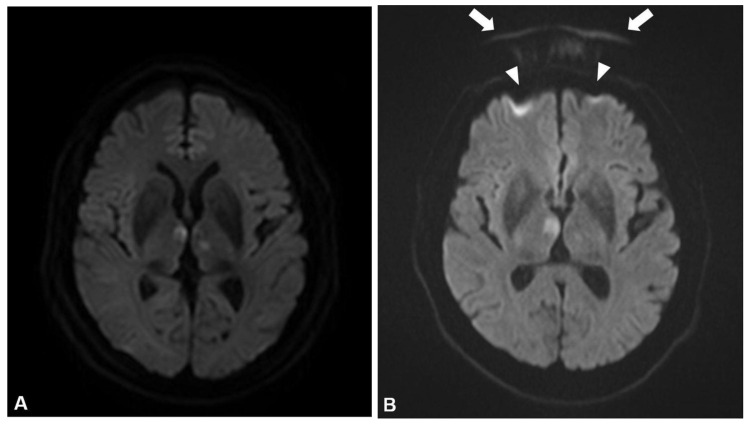
Examples of image quality evaluation of clinical DWI from stroke patients. This patient had a bilateral thalamic acute infarction. (**A**) A b-1000 image with ideal image quality. The image demonstrated the anatomical structure and tissue of interest without any artifacts. (**B**) An example of an image of the same patient with a Nyquist ghost artifact and spatial distortion. Nyquist ghost artifact with duplication of the frontal bone in the phase encoding direction (arrows) and mild distortion of both frontal lobes due to a susceptibility artifact (arrowheads). Spatial distortion and the Nyquist ghost artifact were scored as “acceptable” for this image.

The results of image quality assessment, measured ADC values, and repeatability parameters are presented in Table 6. DWI data from MRI scanners fulfilled the ideal quality for all items except for one scan from a 1.5-T machine having mild spatial distortion and a Nyquist ghost artifact (Figure 4, scored acceptable for each item), and one scan from a 3.0-T machine had a Nyquist ghost artifact (acceptable). None of scans had unacceptable item. For the brain parenchyma, the mean ADC value from three MRI scanners ranged 808.6 μm^2^/s to 843.7 μm^2^/s. The repeatability of ADC value measurement for the brain parenchyma showed a wCV ranging from 2.0% and 2.1%, which met the QIBA claim. The CSF, which has relatively low diffusion restriction, also showed good repeatability (2.0% and 2.1%) within the QIBA claims. The long-term reproducibility of ADC value measurement for the brain parenchyma showed good performance with wCV ranging from 1.8% to 1.9%, which met the QIBA claim. The mean ADC value of CSF from four scanners ranged 3011.0 μm^2^/s to 3156.5 μm^2^/s with a wCV ranging from 2.0% to 2.1%. The experimental ADC value of water at 37 °C is known as 3037.7 μm^2^/s, and the difference between the experimental ADC of water and the measured CSF at 37 °C ranged from 3.6 to 118.8 μm^2^/s [24].

## 4. Discussion

In this study, the performance of four MRI scanners and the clinical protocol for DWI acquisition were evaluated using the QIBA diffusion phantom and a standardized cloud-based phantom analysis tool. All performance metrics of the four MRI scanners were within the range of the QIBA profile claim without additional calibration procedures. These results validated the accuracy and repeatability of DWI acquisition and ADC measurement with these four MRI scanners. The clinical brain protocols also showed excellent accuracy and repeatability in ADC measurement in the phantom imaging analysis. This indicates that our clinical brain protocols can be applied to patient scanning, utilizing ADC as an imaging biomarker in both clinical practice and trials. Indeed, the DWI from 17 patients acquired for a clinical trial fulfilled ideal quality for most of the QC check items and showed excellent long-term repeatability according to the QIBA claims. 

In the present study, we used the QIBA diffusion phantom, which is commercially available along with the cloud-based standardized phantom analysis tool. Recently, QIBA developed a standardized diffusion phantom, allowing any institution or researcher to purchase both the standardized phantom and the analysis tool. This study might be the first published study to report experience with standardization of DWI using both the QIBA diffusion phantom and cloud-based analysis tool. Unlike individually developed DWI phantoms, the QIBA phantom provides standardized materials ready for use by individual institutions. Previous studies validated DWI as an imaging biomarker at individual institutions using separately developed phantoms. However, the use of different validation tools posed a challenge in applying a consistent validation method across various institutions. The standardized QIBA phantom facilitates a common validation method across multiple institutions, contributing to the establishment of DWI as a reliable imaging biomarker.

In our study, we validate MRI equipment using QIBA phantom protocol and clinical brain protocols. The notable differences between the QIBA phantom protocol and clinical brain protocols were b-values (0, 500, 1000, 1500, and 2000 s/mm^2^ for the QIBA phantom protocol vs. 0 and 1000 s/mm^2^ for the clinical brain protocol), repetition time (TR) (8000 ms vs. 3000 ms), and ADC map creation method (log-linear model vs. mono-exponential model). In general, clinical acquisition protocols for brain DWI in many institutions are optimized for a clinical setting, prioritizing shorter acquisition times while maintaining acceptable image quality. Clinical protocols adopt a shorter TR and TE with fewer b-values and NEX than the QIBA phantom protocol. DWI acquisition with higher NEX and more b-values can increase the SNR of DWI and accuracy of ADC measurement. Nevertheless, our study demonstrated that the clinical brain protocols also exhibited comparably good reproducibility in ADC values, meeting the QIBA claims.

The validated clinical brain protocols also demonstrated excellent image quality and high long-term repeatability when applied to patients in a clinical trial. To ensure reliable DWI scanning in patients, periodic QA procedures are crucial [25]. Image QA encompasses a comprehensive quality management process. This involves the validation of MRI equipment performance and clinical acquisition protocols using a phantom, as well as evaluating image quality on actual patient DWI scans. [16]. According to the QIBA profile, periodic QA should be performed, especially in clinical trials using DWI and ADC measurement as quantitative biomarkers. In this study, the entire quality assessment process took approximately 30 min, covering the preparation of the phantom and MRI equipment, image acquisition, and analysis report generation. Considering the relatively short time of the QA process, implementing quality control with the QIBA phantom and profile ensures machine performance and proves advantageous in terms of efficiency. 

Our study has several limitations. First, the number of MRI scanners and clinical brain protocols was relatively small. Several previous studies have demonstrated the accuracy of ADC measurements across various scanners in large number. However, to the best of our knowledge, this study is the first to show the complete process of standardizing DWI using a commercially available phantom. To establish DWI as a widely-used quantitative biomarker, it is crucial to implement a standardized validation process for MRI scanners using the same phantom. Therefore, further studies with a larger number of MRI scanners and protocols across multiple institutions are necessary. Secondly, we applied the validated clinical protocol for the brain in a small number of patients. Different intrinsic biophysical tissue properties of various organs may affect the ADC values and its repeatability. Thus, further study might be necessary for large number of patient and other various organs to validate the DWI as quantitative imaging biomarker. 

In conclusion, we demonstrated a whole standardization process for DWI from validation of MRI equipment performance and clinical brain protocols to application for patient scanning. This study demonstrated the robustness of DWI with high accuracy and repeatability across diverse MRI equipment and clinically optimized protocols, which is in accordance with the QIBA claims.

## Figures and Tables

**Table 1:** Image acquisition parameters for QIBA phantom protocol.

Parameters	QIBA Phantom Protocol
Field strength (T)	1.5 or 3.0 T
Receiver Coil	Head coil
Sequence	DWI EPI
Slice orientation	Axial
FOV	220 × 220 mm
Acquired Voxel size	1.72 × 1.72 × 4 mm
Acquired Matrix (frequency × phase)	128 × 128
Recon voxel size	0.86 × 0.86 × 4 mm
Recon Matrix	128 × 128 to 256 × 256
Parallel imaging acceleration	factor = 2
Phase encode direction	Anterior-posterior
Frequency encode direction	Right-Left
Oversampling	Off
Number of slices	25
Packages	1
Slice thickness	4 mm
Slice gap	1 mm
B0 Shim	Best quality volume shim
B1 Shim	Off or default
Scan mode	Multislice
Technique	Spin echo
Fast imaging mode	Echo planar imaging
Shot mode	Single shot
Echoes	1
Partial echo	Off
TE	shortest
Flip angle	90 deg
TR	8000 ms
Half scan factor	≥0.75
Water fat shift	Minimum
Fat suppression	STIR
Diffusion encoding directions	Three orthogonal
b-value	0, 500, 1000, 1500, 2000
Average high b-value	Off
Gradient mode	Maximum
NEX	2
Preparation phases	Full prep
Geometry phases	Default
Bandwidth in frequency-direction	Maximum

Abbreviations: DWI, Diffusion-weighted imaging; EPI, Echo planar imaging; FOV, Field of view; NEX, number of excitations; QIBA, Quantitative imaging biomarker alliance; STIR, Short TI inversion recovery; TE, Echo time; TR, Repetition time.

**Table 2:** Specifications for brain DWI scan protocols by QIBA and institution.

Parameters	QIBA Clinical Brain Protocol Requirement	Institution Clinical Brain Protocol for			
		GE SIGNA Architect	Philips Ingenia CX	Siemens MAGNETOM Vida	Siemens Avanto
Field strength (T)	1.5 or 3.0 T	3.0 T	3.0 T	3.0 T	1.5 T
Acquisition sequence	SS-EPI	SS-EPI	SS-EPI	SS-EPI	SS-EPI
Receiver Coil	Ideal ^∫^: 32 channel head array coil	16- or 32-channel head coil	16- or 32-channel head coil	16- or 32-channel head coil	16- or 32-channel head coil
	Target ^∬^: 8–32 channel head array coil				
	Acceptable ^∮^: 8 channel head array coil				
Fat suppression	On	STIR	STIR	STIR	STIR
Number of b-values	Ideal: >3 (including one b = 0–50; one 450–550 s/mm^2^; and one at highest b-value)	2	2	2	2
	Target/Acceptable: 2 (including b = 0–50 s/mm^2^ and at highest b-value)				
Minimum highest b-value	Ideal/Target: b = 1000 s/mm^2^ Acceptable: b = 850–999 s/mm^2^	1000 s/mm^2^	1000 s/mm^2^	1000 s/mm^2^	1000 s/mm^2^
Diffusion encoding directions	Ideal/Target: >3-orthogonal, combined gradient channels	3-orthogonal, combined gradient channels	3-orthogonal, combined gradient channels	3-orthogonal, combined gradient channels	3-orthogonal, combined gradient channels
	Acceptable: >3-orthogonal, single gradient channels				
Slice thickness	Ideal: <4 mm	5 mm	5 mm	5 mm	5 mm
	Target: 4–5 mm				
	Acceptable: 5 mm				
Gap thickness	Ideal/Target: 0–1 mm	2 mm	2 mm	2 mm	2 mm
	Acceptable: 1–2 mm				
Field-of-view	220–240 mm	250 × 250 mm	250 × 250 mm	250 × 250 mm	250 × 250 mm
Acquired Matrix (frequency × phase)	Ideal/Target: (160–256) × (160–256), or 1.5–1 mm in-plane resolution	128 × 128	128 × 128	128 × 128	128 × 128
	Acceptable: 128 × 128, or 1.7 mm in-plane resolution				
Plane orientation	Transversal-axial	Transversal-axial	Transversal-axial	Transversal-axial	Transversal-axial
Phase-encode/frequency-encode direction	Anterior-Posterior/Right-Left	Anterior-Posterior /Right-Left	Anterior-Posterior /Right-Left	Anterior-Posterior /Right-Left	Anterior-Posterior/Right-Left
Number of averages	Ideal/Target: ≥2	1	1	1	2
	Acceptable:1				
Half-scan factor	Acceptable/Target: >0.65	0.811	0.811	0.811	0.811
In-plane parallel imaging acceleration factor	Ideal: 2–3	factor = 2.5	factor = 2.5	factor = 2.5	factor = 2.5
	Acceptable/Target: 2				
TR	Ideal: >5000 ms	3000 ms	3000 ms	3000 ms	3000 ms
	Acceptable/Target: 3000–5000 ms				
TE	Ideal: <60 ms	<60 ms	<60 ms	<60 ms	<60 ms
	Target: minimum TE				
	Acceptable: <120 ms				
Receiver Bandwidth	Ideal/Target: maximum possible in frequency encoding direction (minimum echo spacing)	Maximum	Maximum	Maximum	Maximum
	Acceptable: >1000 Hz/voxel				

Abbreviations: SS-EPI, Single-Shot Echo Planar Imaging; STIR, Short TI inversion recovery; TE, Echo time; TR, Repetition time. ^∫^ Ideal: Meeting this specification may require extra effort or non-standard hardware or software, but is expected to provide better results than meeting the target. ^∬^ Target: Meeting this specification is achievable with reasonable effort and adequate equipment, and is expected to provide better results than meeting the acceptable specification. ^∮^ Acceptable: Actors that shall meet this specification to conform to this profile.

**Table 3:** Definition of performance metrics using phantom imaging and QIBA claims according to QIBA DWI profile.

Performance Metrics		Definition	QIBA Claim
Bias in ADC measurement	ADC bias = μ − DC_true_; or % bias = 100% $\frac{\mu -{DC}_{true}}{{DC}_{true}}$	μ: mean ADC (mm^2^/s) within the ROI DC_true_: ADC value for 0% PVP = 1.1 × 10^−3^ mm^2^/s	Central measurement tube (0% PVP) ≤ 4%
Repeatability	RC = 2.77 ∙ σ_ω_ wCV = 100% $\frac{\sigma \omega }{\mu }$	σ_ω_: standard deviation	short-term RC < 1.5 × 10^−5^ mm^2^/s = 0.015 < μm^2^/s (wCV < 0.5%) long-term RC < 6.5 × 10^−5^ mm^2^/s = 0.065 < μm^2^/s (wCV < 2.2%)
Linearity	μ = *β*0 + *β*1 DC_true_		R-squared (R^2^) of the linear model fit > 0.90 95% CI for the slope within the interval 0.95 to 1.05.
b-value dependence	ADC b-value dependence = 100% $\frac{{ADC}_{bmin,b2}-{ADC}_{bmin,b1}}{{ADC}_{bmin,b1}}$	b_min_ = b0	Maximum difference between any of ADC derived from variable b-values to their average ≤ 2% for central tube
Random measurement error	Random measurement error = 100% $\frac{\sigma }{\mu }$		≤2% for central tube
SNR	SNR = $\frac{Spatial\;mean\;ROI\;on\;Signal\;Image}{Spatial\;mean\;of\;ROI\;on\;Noise\;image}$		b = 0 SNR ≥ 45

Abbreviations: ADC, Apparent diffusion coefficient; RC, Repeatability coefficient; Signal-to-noise ratio; wCV, within-subject coefficient of variation.

**Table 4:** Performance metrics of MRI scanners using phantom imaging acquired using the QIBA phantom protocol.

Performance Metrics		QIBA Claims	GE SIGNA Architect	Philips Ingenia CX	Siemens MAGNETOM Vida	Siemens Avanto
Category	Metric					
Accuracy	ADC bias (%)	Abs () ≤ 3.6	−0.4%	−2.3%	−2.1%	−1.5%
Repeatability	RC	<15 μm^2^/s	3.5 μm^2^/s	8.5 μm^2^/s	0.3 μm^2^/s	2.9 μm^2^/s
	wCV	≤0.5%	0.1%	0.3%	0.1%	0.1%
Linearity	Slope	0.95–1.05	0.98	0.98	0.98	1.00
	R^2^	>0.90	0.999	0.999	0.999	1.0
B-value dependence	Max b-value Dependence	≤2%	0.3%	0.6%	0.1%	1.5%
Precision	Random measurement error	≤2%	0.5%	0.7%	0.6%	1.7%
SNR	SNR for 0% water on b-value 0	≥45	105.2	56.0	230.1	117.4

Abbreviations: ADC, Apparent diffusion coefficient; RC, Repeatability coefficient; R^2^, Coefficient of determination; SNR, Signal-to-noise ratio; wCV, within-subject coefficient of variation.

**Table 5:** Performance metrics of clinical brain protocols using phantom imaging acquired by clinical protocols of each MRI scanner.

Performance Metrics		QIBA Claims	GE SIGNA Architect	Philips Ingenia CX	Siemens MAGNETOM Vida	Siemens Avanto
Category	Metric					
Accuracy	ADC bias (%)	Abs () ≤ 3.6	−3.1%	−0.9%	−1.5%	−0.7%
Repeatability	RC	<15 μm^2^/s	2.6 μm^2^/s	1.2 μm^2^/s	2.9 μm^2^/s	3.1 μm^2^/s
	wCV	≤0.5%	0.1%	0%	0.1%	0.1%
Linearity	Slope	0.95–1.05	0.95	1.00	0.97	0.99
	R^2^	>0.90	0.995	0.997	0.997	0.998
B-value dependence ^†^	Max b-value Dependence	≤2%	NA	NA	NA	NA
Precision	Random measurement error	≤2%	0.5%	0.4%	0.4%	0.8%
SNR	SNR for 0% water on b-value 0	≥45	145.6	323.4	380.3	199.7

Abbreviations: ADC, Apparent diffusion coefficient; RC, Repeatability coefficient; R^2^, Coefficient of determination; SNR, Signal-to-noise ratio; wCV, within-subject coefficient of variation. ^†^ Max b-value dependence was omitted in the institutional protocol validation because the protocol consist of two b-values.

**Table 6:** Image quality and repeatability measurement of patients’ imaging.

		GE SIGNA Architect	Philips Ingenia CX	Siemens Avanto
**Image quality evaluation** ^†^				
Number of patients (scans)		N = 3 (3 scans)	N = 14 (17 scans)	N = 17 (35 scans)
Low SNR		3 ± 0	3 ± 0	3 ± 0
Ghost/parallel imaging artifacts		3 ± 0	3 ± 0	3 ± 0
Severe spatial distortion		3 ± 0	3 ± 0	3.0 ± 0.2
Eddy currents		3 ± 0	3 ± 0	3 ± 0
Fat suppression		3 ± 0	3 ± 0	3 ± 0
Motion artefacts		3 ± 0	3 ± 0	3 ± 0
Nyquist ghost		3 ± 0	2.9 ± 0.3	3.0 ± 0.2
**Repeatability evaluation**				
Number of patients		N = 0	N = 3 (6 scans)	N = 14 (28 scans)
White matter	ADC value (μm^2^/s) ^†^	N.A.	807.7 ± 16.7	801.4 ± 16.2
	RC (μm^2^/s) ^‡^	N.A.	46.3	44.9
	wCV (%) ^‡^	N.A.	2.1	2.0
CSF	ADC value (μm^2^/s) ^†^	N.A.	3079.4 ± 63.6	3013.0 ± 59.9
	RC (μm^2^/s) ^‡^	N.A.	176.3	166.1
	wCV (%) ^‡^	N.A.	2.1	2.00
**Reproducibility evaluation**				
Number of patients		N = 3 (3 scans)	N = 14 (17 scans)	N = 17 (35 scans)
White matter	ADC value (μm^2^/s) ^†^	843.7 ± 15.3	813.7 ± 15.4	803.7 ± 15.2
	RC (μm^2^/s) ^‡^	42.5	42.7	42.1
	wCV (%) ^‡^	1.8	1.9	1.9
CSF	ADC value (μm^2^/s) ^†^	3156.5 ± 65.9	3114.7 ± 63.5	3011.0 ± 60.4
	RC (μm^2^/s) ^‡^	182.6	175.9	167.2
	wCV (%) ^‡^	2.1	2.0	2.0

^†^ Data are reported as the mean ± SD; ^‡^ QIBA claims for long-term repeatability, RC < 6.5 × 10^−5^ mm^2^/s = 0.065 < μm^2^/s (wCV < 2).

## Data Availability

The data presented in this study are available on request from the corresponding author.

## Supplementary Materials

The following supporting information can be downloaded at: https://www.mdpi.com/article/10.3390/diagnostics14060583/s1, S1: Supplementary Material 1; S2: Supplementary Material 2; S3: Supplementary Material 3; Figure S1: Supplementary Figure S1; Figure S2: Supplementary Figure S2.
