# Supplementary material for: Whole Process of Standardization of Diffusion-Weighted Imaging: Phantom Validation and Clinical Application According to the QIBA Profile
Source: Diagnostics (Basel). 2024 Mar 9;14(6):583. doi: 10.3390/diagnostics14060583 (PMC10969680; doi:10.3390/diagnostics14060583)
Supplement: Supplementary file 1 [file diagnostics-14-00583-s001.zip › diagnostics-2858129-supplementary.pdf]

Whole process of standardization of diffusion-weighted: Phantom validation and clinical application according to the QIBA profile

Supplementary Material

Supplementary Material 1. NIST/QIBA diffusion phantom (A) and detailed information about the phantom and preparation method for temperature stability (B)

(A)

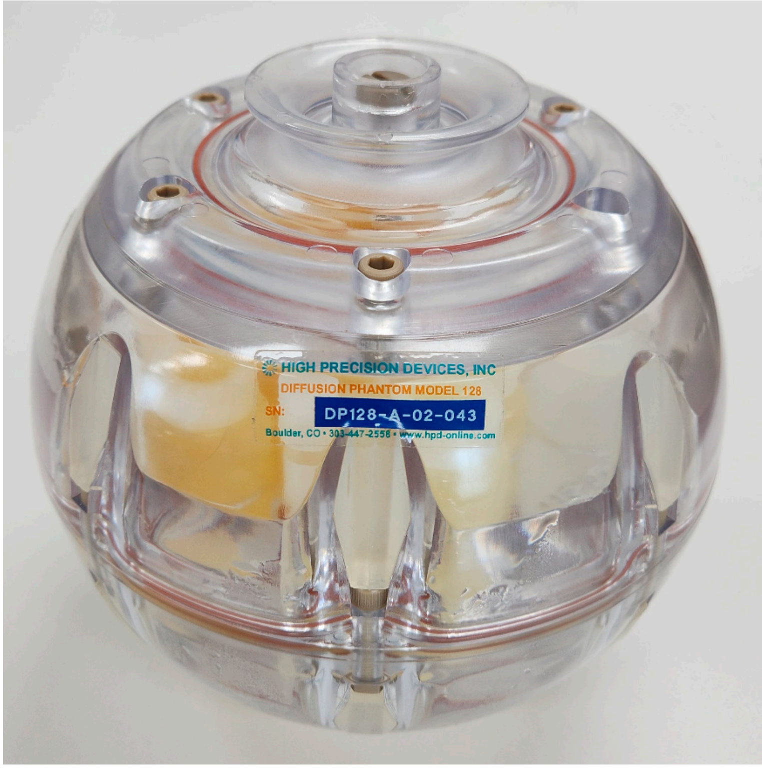

(B) A spherical phantom measuring 194 mm in diameter housing 13 vials (30 mL) filled with varying concentrations of polyvinylpyrrolidone (PVP, [0, 10, 20, 30, 40, and 50% w/w]) was tested. The central vial was filled with pure water (0%), and two arrays of vials containing PVP solution ranging from 0% to 50% consisted of the inner ring and outer ring. PVP solution was used to generate physiologically relevant apparent diffusion coefficient (ADC) values, and a higher concentration of PVP resulted in a

lower ADC value. Considering the temperature sensitivity of diffusion properties, the ADC value of each PVP concentration at 0°C verified by the National Institute of Standards and Technology was used as reference standard. To obtain homogeneity and stability of the phantom temperature during the test, the space between the cylinders was filled with crushed ice and tap water prior to scanning over one hour according to the instructions provided by the Quantitative Imaging Biomarker Alliance. The phantom was maintained at approximately 0°C during the scanning.

Supplementary Material 2. Details of analytical method of the image quality assessment software

The quantitative analysis of DWI was performed by measuring the ADC values from volumes-of-interest (VOIs) derived from circular regions of interest (ROIs) measuring 19.6 mm in diameter on five slices. ADC maps were created from multiple DWI b-value pairs, and for the clinical DWI protocol, b-values of 1000 and 0 s/mm^2 were used. ADC map was created using mono-exponential model. The reference standard for the ADC value of each PVP concentration was the NIST-verified value provided by the QIBA profile. The analytical method used in the commercial software was identical to the standard analysis software provided by the Quantitative Imaging Data Warehouse (QIBA QIDW, rsna.org/qidw). QIBAphan, open-source DWI phantom QC analysis software provided by QIBA QIDW, can be accessed online at https://bit.ly/2QXLo3e. QIBAphan converts QIBA DWI data from classic DICOM format into uniform data structures for generating QC statistics. Users select ROI centers in each slice of the DWIs, and the software automatically generates statistics on the ADC values across the VOIs. QIBAphan provides a QC report that includes processed output ROI statistics and performance metrics in CSV files.

Supplementary Material 3. Details of items for image quality assurance and scale

Low SNR: Visualization of anatomical features in tissues of interest at all b-values was evaluated: unacceptable, poor SNR at all b-values with anatomical features are lost; acceptable, minor deterioration of image without disturbing visualization of anatomical structure; ideal, identification of all anatomical structures with accurate structure

Ghost/parallel imaging artifacts: The presence of the discrete ghosts from extraneous signal sources along the phase-encode direction obscuring the tissue of interest was evaluated: unacceptable, presence of artifact creating erroneous ADC value; acceptable, minor artifact without disturbing assessment of performance parameter; ideal, visualization of anatomical features in tissues of interest and no artifact

Severe spatial distortion: Severe spatial distortions affecting ADC values and the apparent size/shape/volume of tissues of interest were investigated: unacceptable, severe distortion altering apparent size/shape/volume of tissue of interest; acceptable, minor distortion without significant modify of shape of tissue; ideal, no distortion of tissue of interest

Eddy currents: Blur or spatial misalignment between low and high b-value diffusion-weighted imaging (DWI), particularly at the edges of anatomical features, was evaluated: unacceptable, blur of anatomy causes and erroneous measurement of ADC value; acceptable, minor spatial misregistration only affecting the edge of the lesion; ideal, no evidence of blur or spatial misalignment in all images

Fat suppression: Superposition of unsuppressed fat signal on the tissue of interest was assessed: unacceptable, unsuppressed fat signal spatially shifted obscuring the tissue of interest and renders ADC meaningless in tissue superimposed by a residual fat signal; acceptable, minor detrimental chemical shift artifacts not affecting tissue of interest; ideal, complete suppressed fat signal onto tissue of interest

Motion artefacts: The presence of cerebrospinal fluid (CSF) pulsation in the ventricles or cardiac pulsation near large vessels and the brainstem was assessed: unacceptable, motion artifact contributes to blurring image and erroneous signal leading to unpredictable ADC values; acceptable, minor artifact without disturbing assessment of tissue of interest; ideal, no motion artifact in the image

Nyquist ghost: Duplication of an anatomical structure or distortion of an image occurring in the phase encoding direction was evaluated. Unacceptable, artifact presence of artifact disturbing recognition of anatomical structure and obscure tissue of interest leading to unpredictable ADC values; Acceptable, minor artifact without disturbing assessment of performance parameter; Ideal, Visualization of anatomical features in tissues of interest and no artifact

Supplementary Figure S1. The phantom data analysis reports using QIBA acquisition protocol. The report comprises a table presenting ADC VOI (Volume of Interest) statistics and graphs include ADC value vs. Axial position showcasing the ADC values of each vial at specific axial positions, NIST ADC value vs. measured ADC value comparing NIST (National Institute of Standards and Technology) ADC values with measured ADC values to assess the correlation between the two values, and NIST ADC value vs. within-subject Coefficient of Variation (wCV) displays the relationship between NIST ADC values and within-subject Coefficient of Variation (wCV). These analyses are conducted for four different MRI systems: (A) Architect, (B) Ingenia, (C) Vida, and (D) Avanto.

ADC VOI Statistics

Label	Contents	# Voxels	NIST Value	Mean	Bias	Bias Percent	Max B-Value Dependence	SNR B0	SNR B500	SNR B1000	SNR B1500	SNR B2000	RC	Random Error	wCV
			um^2/s	um^2/s	um^2/s	um^2/s	% (associated b-value pair)						um^2/sec	%	%
Vial 1 - Limits					*abs() <= 40*	*abs() <= 3.6*	*< 2*	*>= 50*					*<= 15*	*<= 3.6*	*<= 0.5*
①	water	12064	1127	1122.2	-4.8	-0.4	0.31 (500, 2000)	105.2	97.9	86.0	79.1	63.2	3.51	0.48	0.11
②	water	12064	1127	1138.2	11.2	1.0	0.21 (500, 1500)	53.2	64.5	65.5	66.5	60.5	3.73	0.88	0.12
③	PVP50	12064	128	158.4	30.4	23.7	32.50 (500, 2000)	39.5	68.3	81.8	84.7	86.4	4.63	6.51	1.06
④	PVP40	12064	248	283.8	35.8	14.5	9.19 (500, 2000)	74.5	105.4	117.0	118.0	110.4	4.04	2.05	0.51
⑤	PVP30	12064	403	419.6	16.6	4.1	0.64 (500, 1000)	107.3	152.7	165.5	171.2	167.5	1.54	0.95	0.13
⑥	PVP20	12064	607	600.6	-6.4	-1.1	3.97 (500, 2000)	122.5	176.1	183.1	180.4	158.6	2.09	0.61	0.13
⑦	PVP10	12064	843	844.4	1.4	0.2	2.93 (500, 2000)	107.1	150.7	137.4	129.6	115.9	0.54	0.48	0.02
⑧	PVP50	12064	128	115.8	-12.2	-9.5	7.96 (1000, 2000)	63.4	122.8	151.2	163.0	163.2	3.56	5.99	1.11
⑨	PVP40	12064	248	268.3	20.3	8.2	9.66 (500, 2000)	85.0	126.5	153.7	161.7	163.0	1.97	1.97	0.27
⑩	PVP30	10556	403	431.8	28.8	7.1	1.87 (500, 2000)	107.7	134.9	143.5	150.5	148.0	0.68	0.92	0.06
⑪	PVP20	12064	607	603.5	-3.5	-0.6	2.02 (500, 2000)	80.0	75.3	61.4	57.0	54.5	10.59	0.86	0.63
⑫	PVP10	11310	843	853.5	10.5	1.3	0.13 (500, 1500)	61.6	55.6	51.2	48.3	49.9	5.85	0.73	0.25
⑬	water	12064	1127	1136.4	9.4	0.8	0.66 (500, 2000)	117.4	133.0	120.8	99.6	77.4	7.10	0.49	0.23

ADC Value vs Axial Position

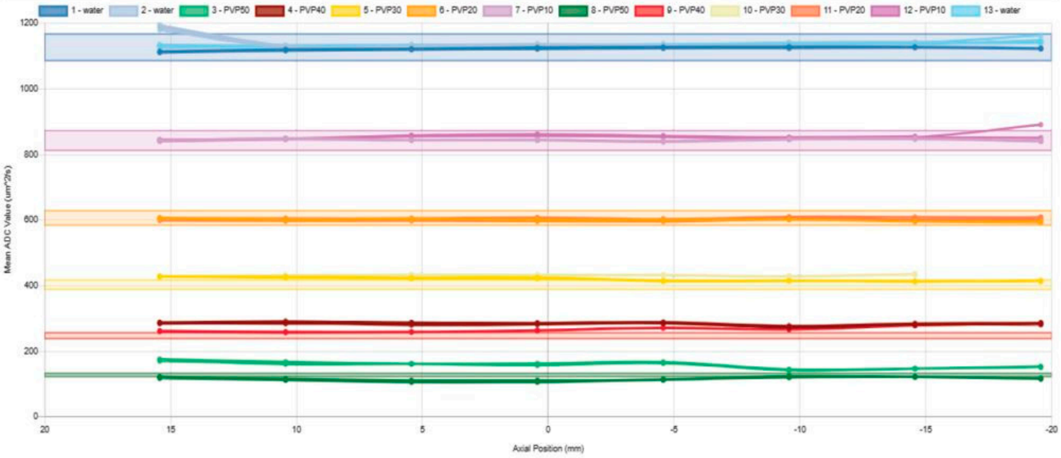

NIST ADC Value vs Measured ADC Value

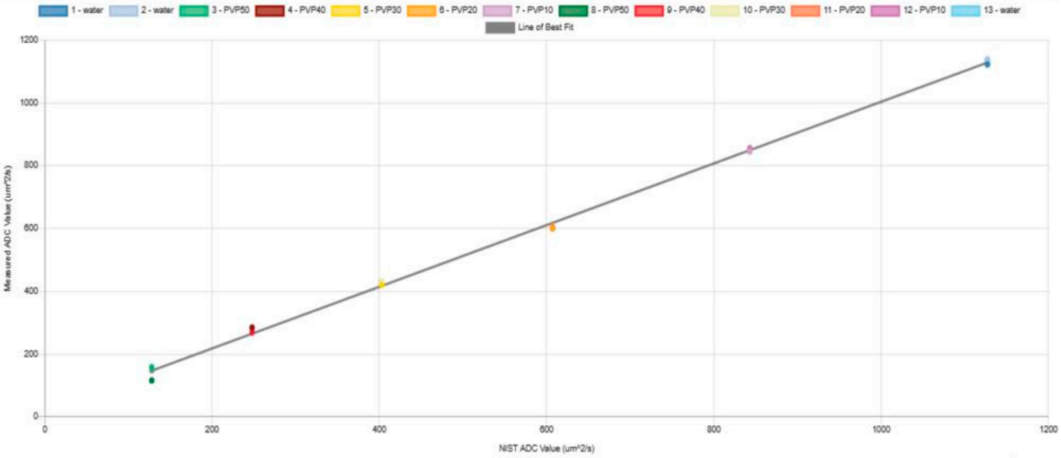

Best Fit Slope: 0.982 R^2: 0.998

NIST ADC Value vs wCV

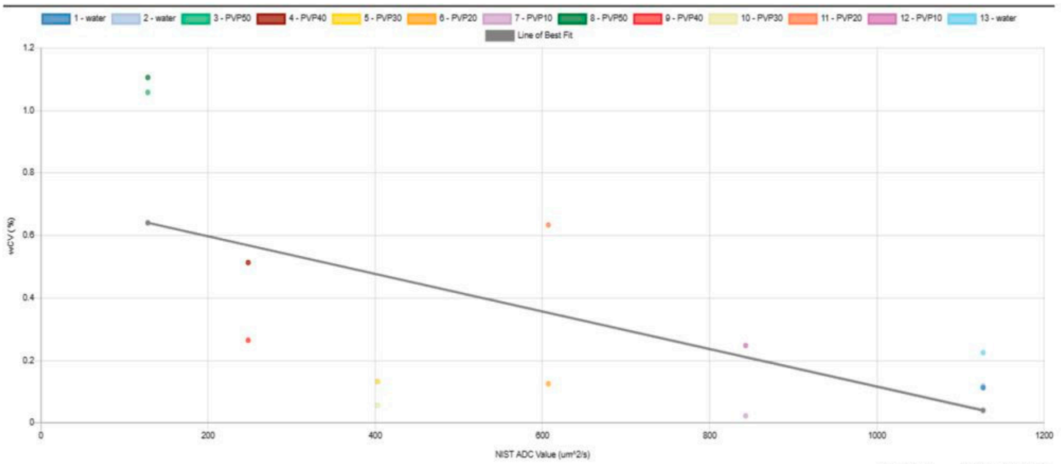

Best Fit Slope: -0.001 R^2: 0.385

ADC VOI Statistics

Label	Contents	# Voxels	NIST Value	Mean	Bias	Bias Percent	Max B-Value Dependence	SNR B0	SNR B500	SNR B1000	SNR B1500	SNR B2000	RC	Random Error	wCV
			um^2/s	um^2/s	um^2/s	um^2/s	% (associated b-value pair)						um^2/sec	%	%
Vial 1 - Limits					*abs() <= 40*	*abs() <= 3.6*	*< 2*	*>= 50*					*<= 15*	*<= 3.6*	*<= 0.5*
1	water	12064	1127	1101.5	-25.5	-2.3	0.60 (500, 2000)	56.0	63.7	69.4	68.1	58.6	8.51	0.67	0.28
2	water	12064	1127	1112.0	-15.0	-1.3	1.99 (500, 2000)	48.2	52.3	51.9	50.7	46.5	2.23	0.56	0.07
3	PVP50	12064	128	153.7	25.7	20.1	21.59 (500, 2000)	47.3	53.1	56.0	56.7	59.7	4.22	3.06	0.99
4	PVP40	12064	248	245.3	-2.7	-1.1	1.01 (500, 1500)	53.9	60.1	63.9	66.9	68.6	4.34	1.77	0.64
5	PVP30	12064	403	399.5	-3.5	-0.9	7.46 (500, 2000)	57.3	65.2	68.2	69.5	71.8	5.44	0.80	0.49
6	PVP20	12064	607	593.4	-13.6	-2.2	1.26 (500, 2000)	51.5	56.0	58.1	59.0	59.2	4.08	0.56	0.25
7	PVP10	12064	843	832.3	-10.7	-1.3	1.72 (500, 2000)	59.1	62.1	64.0	63.0	61.7	2.28	0.47	0.10
8	PVP50	12064	128	133.5	5.5	4.3	22.20 (500, 2000)	44.8	49.4	52.1	54.0	56.0	3.10	3.62	0.83
9	PVP40	12064	248	242.0	-6.0	-2.4	1.19 (500, 1000)	42.2	49.4	52.0	54.1	55.4	3.83	2.27	0.57
10	PVP30	12064	403	394.1	-8.9	-2.2	2.65 (500, 2000)	50.5	53.3	56.1	57.5	59.5	4.51	0.79	0.41
11	PVP20	12064	607	591.5	-15.5	-2.6	1.65 (500, 2000)	50.0	53.4	56.0	58.0	60.8	6.07	0.57	0.37
12	PVP10	12064	843	825.7	-17.3	-2.1	0.45 (500, 1500)	43.0	44.9	46.3	47.9	48.1	4.59	0.41	0.20
13	water	12064	1127	1119.0	-8.0	-0.7	0.80 (500, 2000)	54.2	58.9	62.1	64.0	60.0	8.45	0.49	0.27

ADC Value vs Axial Position

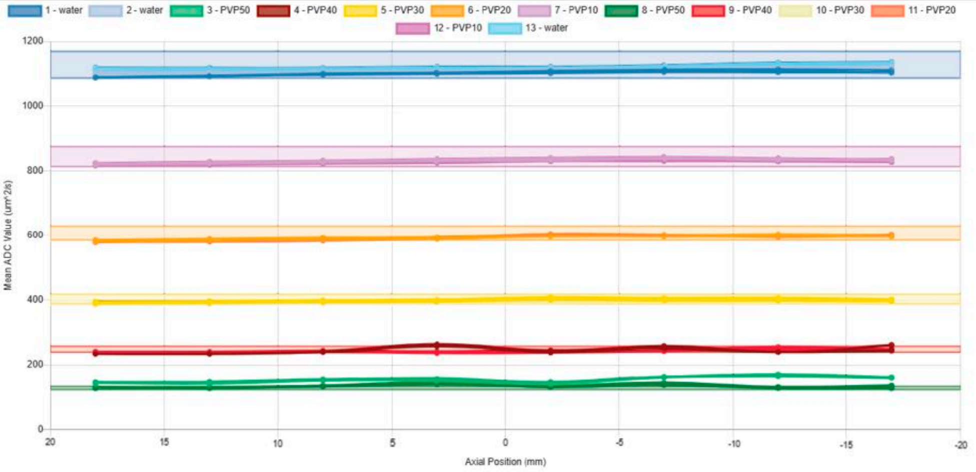

NIST ADC Value vs Measured ADC Value

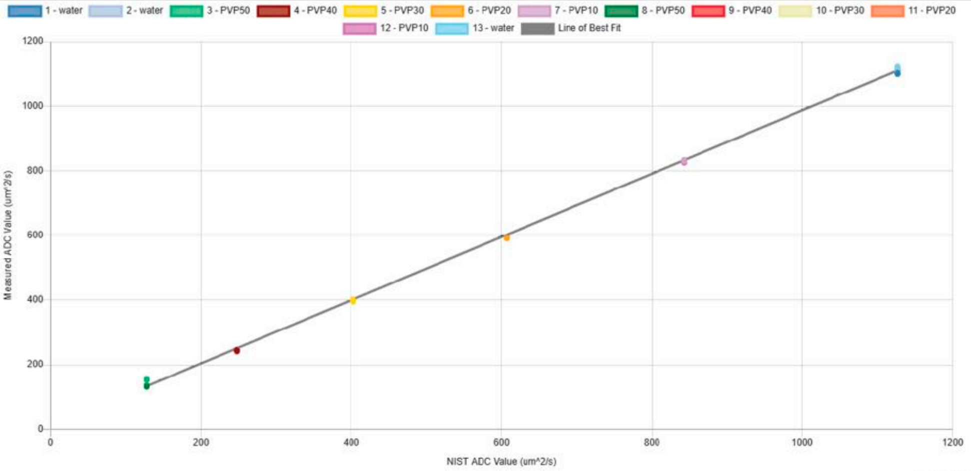

Best Fit Slope: 0.978 R^2: 0.999

NIST ADC Value vs wCV

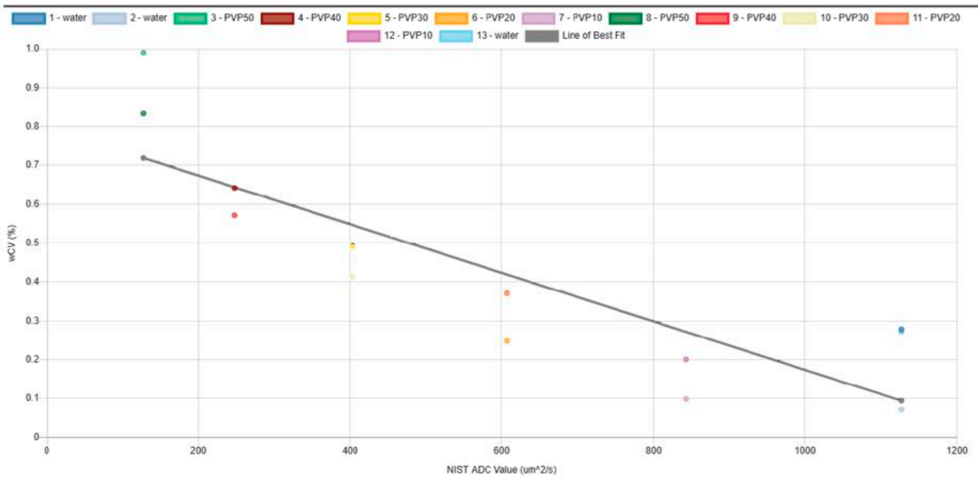

Best Fit Slope: -0.001 R^2: 0.733

ADC VOI Statistics

Label	Contents	# Voxels	NIST Value	Mean	Bias	Bias Percent	Max B-Value Dependence	SNR B0	SNR B500	SNR B1000	SNR B1500	SNR B2000	RC	Random Error	wCV
			um^2/s	um^2/s	um^2/s	um^2/s	% (associated b-value pair)						um^2/sec	%	%
Vial 1 - Limits					*abs() <= 40*	*abs() <= 3.6*	*< 2*	*>= 50*					*<= 15*	*<= 3.6*	*<= 0.5*
1	water	12064	1127	1103.4	-23.6	-2.1	0.06 (500, 2000)	230.1	181.2	141.7	103.7	64.1	0.31	0.61	0.01
2	water	12064	1127	1103.9	-23.1	-2.1	1.10 (500, 2000)	107.1	94.3	83.4	64.1	46.9	3.92	0.86	0.13
3	PVP50	12064	128	128.9	0.9	0.7	6.53 (500, 2000)	86.7	125.5	137.2	139.1	133.7	3.37	4.24	0.94
4	PVP40	12064	248	243.4	-4.6	-1.8	0.56 (500, 1000)	154.9	217.0	216.3	202.9	191.1	1.50	1.31	0.22
5	PVP30	12064	403	407.2	4.2	1.0	1.57 (500, 2000)	219.9	272.8	242.2	216.9	184.0	2.29	0.73	0.20
6	PVP20	12064	607	604.7	-2.3	-0.4	0.59 (500, 2000)	217.9	267.8	241.2	201.4	160.7	1.42	0.52	0.08
7	PVP10	12064	843	839.3	-3.7	-0.4	0.65 (500, 2000)	160.2	173.7	146.2	116.5	81.7	2.14	0.63	0.09
8	PVP50	12064	128	134.5	6.5	5.1	9.51 (500, 2000)	81.6	120.0	122.0	124.4	120.5	1.54	4.19	0.41
9	PVP40	12064	248	256.7	8.7	3.5	1.29 (500, 2000)	197.6	244.1	252.2	229.5	220.4	1.49	0.94	0.21
10	PVP30	12064	403	402.3	-0.7	-0.2	0.30 (500, 1000)	209.2	251.0	253.5	238.4	216.7	1.17	0.65	0.10
11	PVP20	12064	607	610.1	3.1	0.5	0.52 (500, 2000)	179.5	206.0	185.9	168.3	142.3	0.67	0.47	0.04
12	PVP10	12064	843	843.7	0.7	0.1	0.44 (500, 1500)	101.8	105.4	103.7	97.1	82.4	2.26	0.54	0.10
13	water	12064	1127	1135.4	8.4	0.7	1.28 (500, 2000)	142.2	122.9	99.9	73.0	45.4	4.73	0.80	0.15

ADC Value vs Axial Position

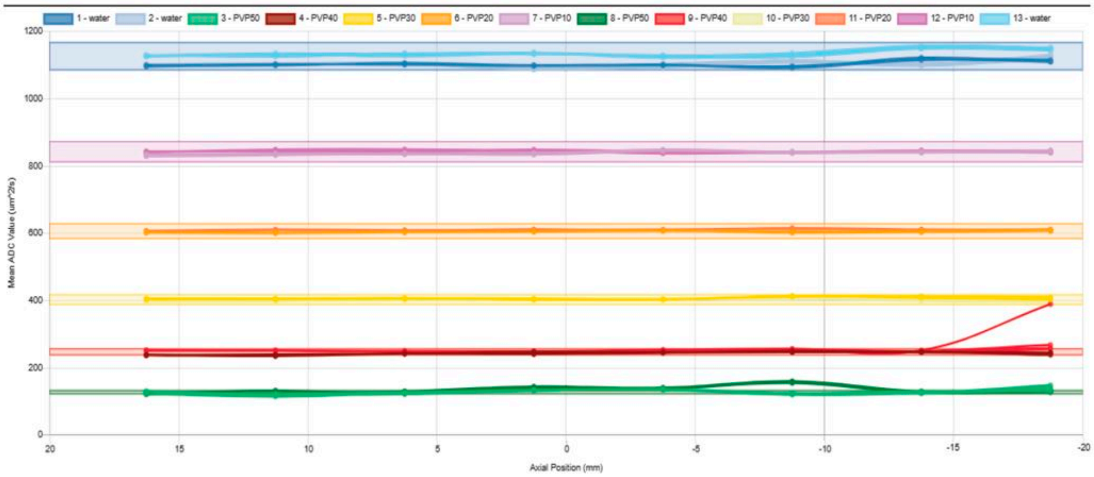

NIST ADC Value vs Measured ADC Value

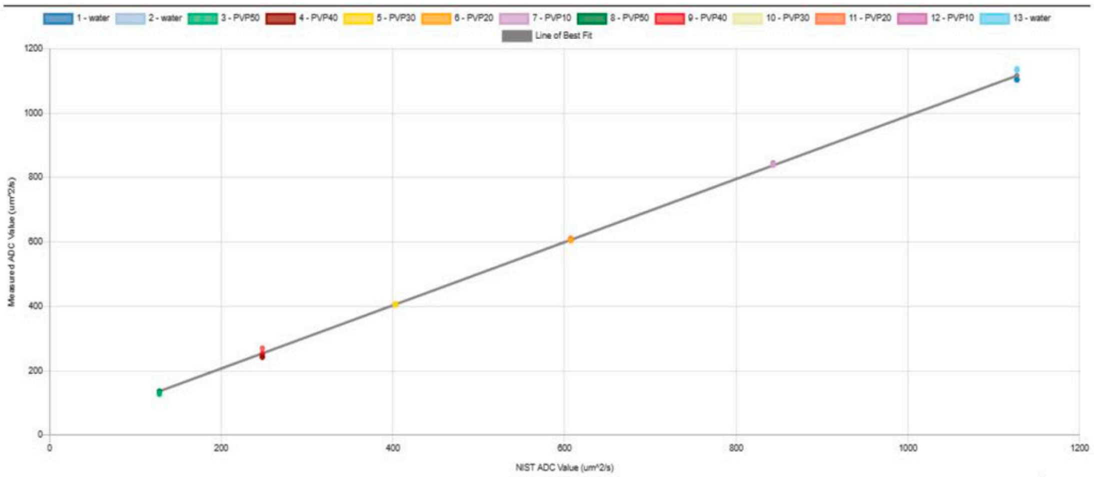

Best Fit Slope: 0.980 R^2: 0.999

NIST ADC Value vs wCV

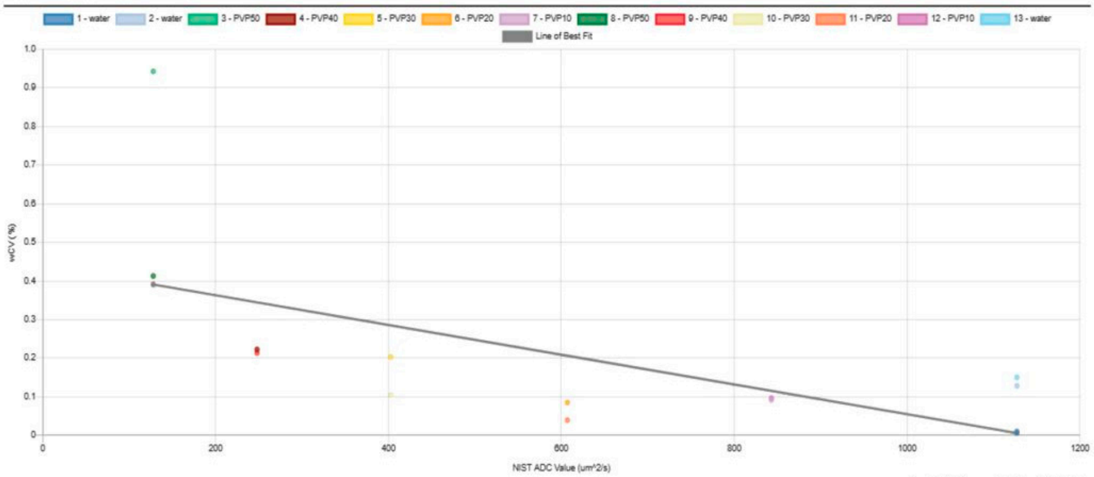

Best Fit Slope: -0.000 R^2: 0.359

ADC VOI Statistics

Label	Contents	# Voxels	NIST Value	Mean	Bias	Bias Percent	Max B-Value Dependence	SNR B0	SNR B500	SNR B1000	SNR B1500	SNR B2000	RC	Random Error	wCV
			um^2/s	um^2/s	um^2/s	um^2/s	% (associated b-value pair)						um^2/sec	%	%
Vial 1 - Limits					*abs() <= 40*	*abs() <= 3.6*	*< 2*	*>= 50*					*<= 15*	*<= 3.6*	*<= 0.5*
1	water	14112	1127	1110.4	-16.6	-1.5	1.47 (500, 1500)	117.4	115.1	65.1	38.2	22.1	2.89	1.73	0.09
2	water	14112	1127	1123.7	-3.3	-0.3	0.60 (500, 1000)	144.9	136.5	79.2	45.4	26.9	5.01	1.43	0.16
3	PVP50	14112	128	122.0	-6.0	-4.7	8.41 (500, 1500)	44.9	70.5	69.8	63.4	60.6	0.92	10.08	0.27
4	PVP40	14112	248	232.6	-15.4	-6.2	2.36 (500, 2000)	75.6	116.4	106.6	95.6	80.9	2.11	3.27	0.33
5	PVP30	13671	403	398.4	-4.6	-1.1	1.37 (500, 2000)	87.3	121.7	97.6	84.3	65.4	1.65	2.02	0.15
6	PVP20	14112	607	607.2	0.2	0.0	1.31 (500, 2000)	107.8	137.2	101.3	76.3	57.5	1.39	1.39	0.08
7	PVP10	14112	843	840.0	-3.0	-0.4	0.39 (1000, 2000)	118.1	131.2	87.3	60.1	40.7	2.13	1.31	0.09
8	PVP50	14112	128	123.1	-4.9	-3.8	3.03 (500, 1000)	53.1	86.4	82.3	77.5	70.2	4.30	8.46	1.26
9	PVP40	14112	248	236.6	-11.4	-4.6	7.37 (500, 2000)	96.0	147.6	128.6	115.0	102.8	2.04	2.64	0.31
10	PVP30	14112	403	392.5	-10.5	-2.6	3.03 (500, 2000)	119.5	164.6	131.6	112.9	93.6	0.59	1.45	0.05
11	PVP20	13671	607	612.4	5.4	0.9	2.26 (500, 2000)	120.2	154.5	116.5	88.1	64.3	2.78	1.23	0.16
12	PVP10	13671	843	836.3	-6.7	-0.8	0.43 (1000, 2000)	119.2	136.3	95.5	62.7	43.4	2.04	1.27	0.09
13	water	14112	1127	1109.8	-17.2	-1.5	0.50 (1000, 2000)	154.9	151.2	88.7	51.6	30.8	2.43	1.25	0.08

ADC Value vs Axial Position

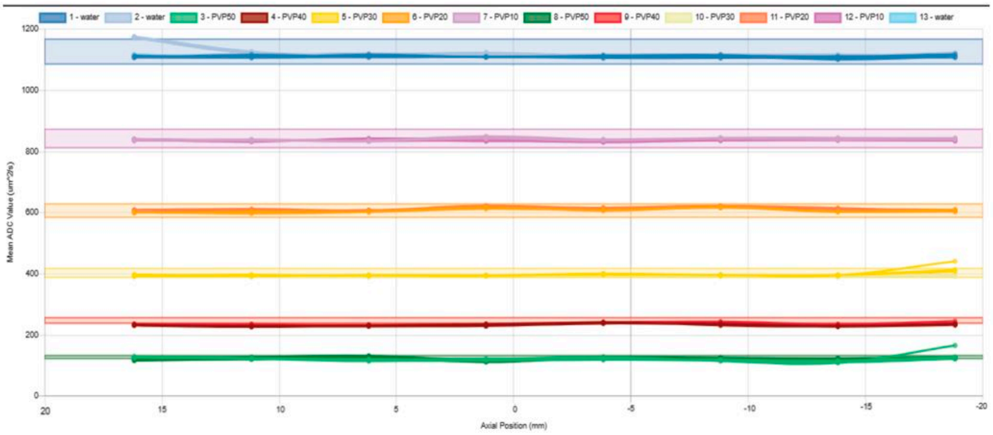

NIST ADC Value vs Measured ADC Value

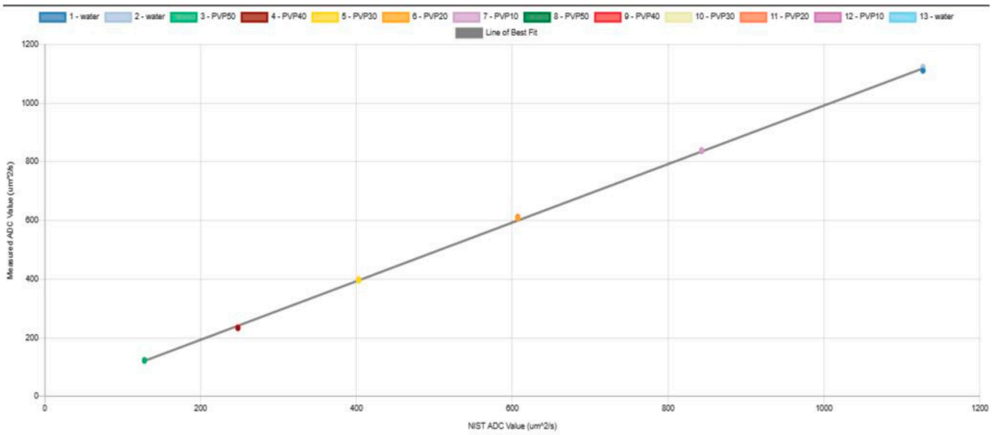

Best Fit Slope: 0.998 R^2: 1.000

NIST ADC Value vs wCV

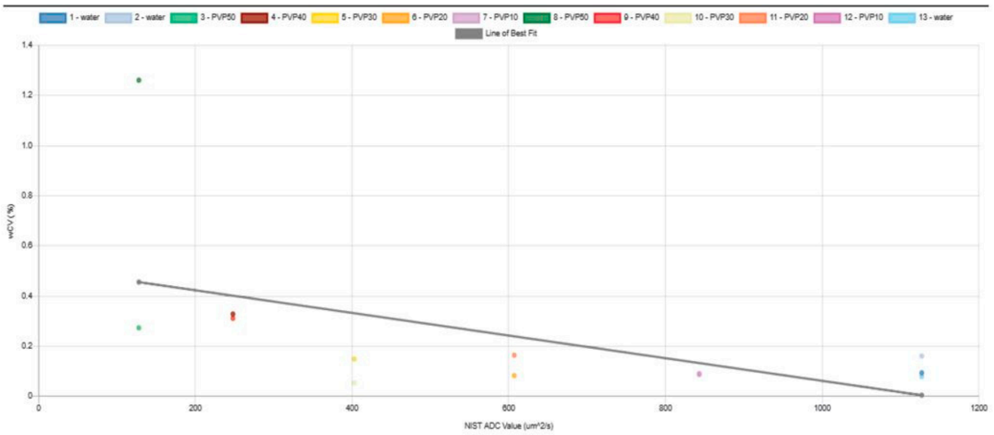

Best Fit Slope: -0.000 R^2: 0.286

Supplementary Figure S2. The phantom data analysis reports using the clinical acquisition protocols.

The report comprises a table presenting ADC VOI (Volume of Interest) statistics and graphs include ADC value vs. Axial position showcasing the ADC values of each vial at specific axial positions, NIST ADC value vs. measured ADC value comparing NIST (National Institute of Standards and Technology) ADC values with measured ADC values to assess the correlation between the two values, and NIST ADC value vs. within-subject Coefficient of Variation (wCV) displays the relationship between NIST ADC values and within-subject Coefficient of Variation (wCV). These analyses are conducted for four different MRI systems: (A) Architect, (B) Ingenia, (C) Vida, and (D) Avanto.

ADC VOI Statistics

Label	Contents	# Voxels	NIST Value	Mean	Bias	Bias Percent	Max B-Value Dependence	SNR B0	SNR B1000	RC	Random Error	wCV
			um^2/s	um^2/s	um^2/s	um^2/s	% (associated b-value pair)			um^2/sec	%	%
Vial 1 - Limits					*abs() <= 40*	*abs() <= 3.6*	*< 2*	*>= 50*		*<= 15*	*<= 3.6*	*<= 0.5*
①	water	5706	1127	1092.6	-34.4	-3.1		145.6	146.1	2.56	0.51	0.08
②	water	5706	1127	1103.8	-23.2	-2.1		104.0	117.0	1.59	0.57	0.05
③	PVP50	5706	128	201.0	73.0	57.0		95.9	129.2	3.58	4.20	0.64
④	PVP40	5706	248	282.3	34.3	13.8		113.7	136.8	2.60	1.94	0.33
⑤	PVP30	5706	403	401.5	-1.5	-0.4		109.4	123.4	1.07	1.32	0.10
⑥	PVP20	5706	607	570.7	-36.3	-6.0		101.7	110.4	2.48	0.81	0.16
⑦	PVP10	5706	843	806.4	-36.6	-4.3		106.7	110.5	2.03	0.62	0.09
⑧	PVP50	5389	128	107.8	-20.2	-15.8		83.5	111.2	4.45	7.81	1.46
⑨	PVP40	5706	248	240.7	-7.3	-2.9		93.6	107.6	2.78	2.30	0.42
⑩	PVP30	5706	403	423.4	20.4	5.1		99.3	112.9	3.40	1.07	0.29
⑪	PVP20	5706	607	571.2	-35.8	-5.9		84.2	88.6	1.22	0.81	0.08
⑫	PVP10	5706	843	818.1	-24.9	-2.9		69.1	83.8	6.84	1.06	0.30
⑬	water	5706	1127	1110.2	-16.8	-1.5		88.6	93.9	0.39	0.42	0.01

ADC Value vs Axial Position

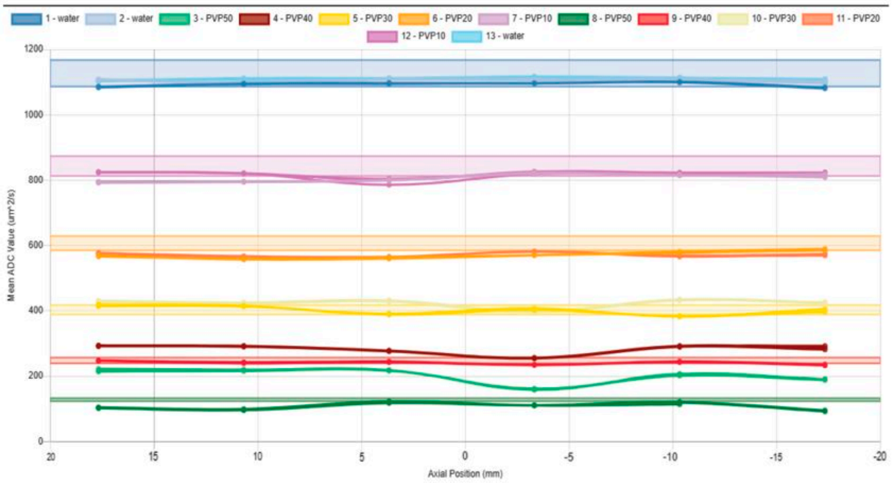

NIST ADC Value vs Measured ADC Value

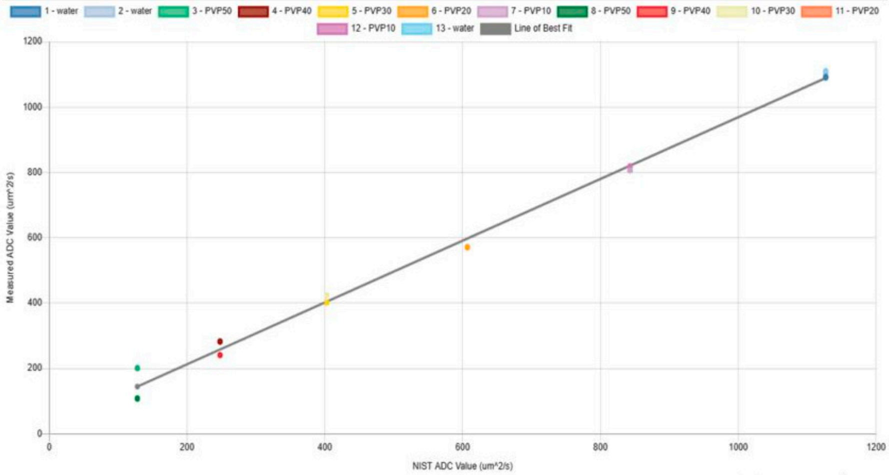

Best Fit Slope: 0.946 R^2: 0.995

NIST ADC Value vs wCV

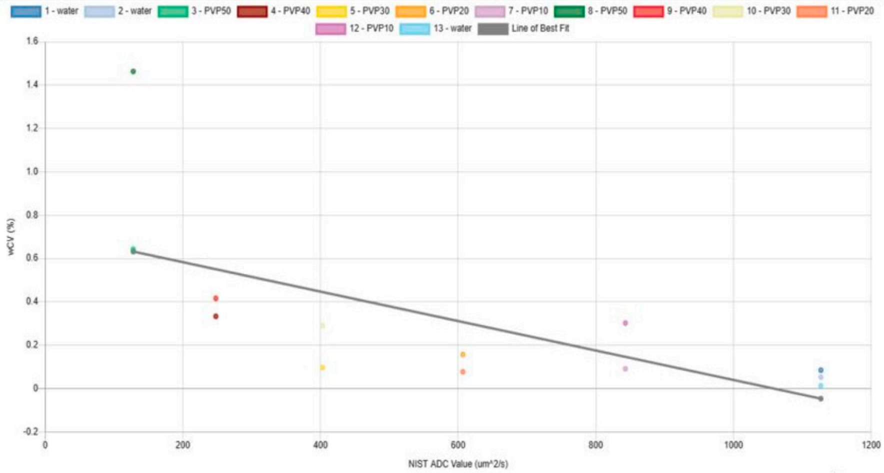

Best Fit Slope: -0.001 R^2: 0.434

ADC VOI Statistics

Label	Contents	# Voxels	NIST Value	Mean	Bias	Bias Percent	Max B-Value Dependence	SNR B0	SNR B1000	RC	Random Error	wCV
			um^2/s	um^2/s	um^2/s	um^2/s	% (associated b-value pair)			um^2/sec	%	%
Vial 1 - Limits					*abs() <= 40*	*abs() <= 3.6*	*< 2*	*>= 50*		*<= 15*	*<= 3.6*	*<= 0.5*
①	water	7608	1127	1117.3	-9.7	-0.9		323.4	277.7	1.20	0.39	0.04
②	water	7608	1127	1088.5	-38.5	-3.4		227.0	196.1	1.35	0.43	0.04
③	PVP50	7608	128	98.0	-30.0	-23.5		141.8	244.2	3.75	7.48	1.39
④	PVP40	7608	248	244.4	-3.6	-1.5		220.7	323.5	1.79	1.94	0.27
⑤	PVP30	7608	403	414.7	11.7	2.9		249.9	320.0	1.59	0.91	0.14
⑥	PVP20	7608	607	611.5	4.5	0.7		279.2	295.3	1.79	0.60	0.11
⑦	PVP10	7608	843	835.7	-7.3	-0.9		285.3	288.7	0.95	0.44	0.04
⑧	PVP50	7608	128	102.9	-25.1	-19.6		140.6	242.9	3.10	6.56	1.09
⑨	PVP40	7608	248	223.4	-24.6	-9.9		193.2	227.2	3.37	1.92	0.54
⑩	PVP30	7608	403	415.0	12.0	3.0		264.6	304.8	1.27	0.86	0.11
⑪	PVP20	7608	607	636.1	29.1	4.8		254.2	283.0	2.88	0.56	0.16
⑫	PVP10	7608	843	869.3	26.3	3.1		102.3	132.1	1.64	0.98	0.07
⑬	water	7608	1127	1114.6	-12.4	-1.1		217.6	193.1	3.20	0.37	0.10

ADC Value vs Axial Position

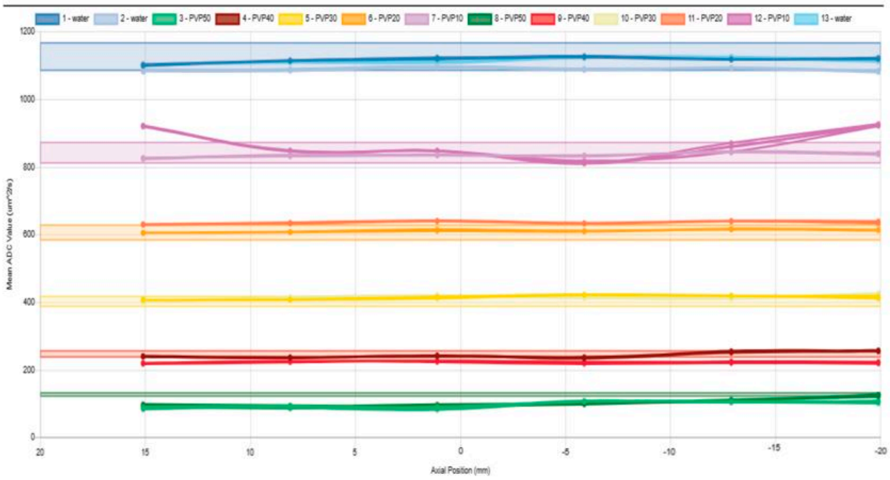

NIST ADC Value vs Measured ADC Value

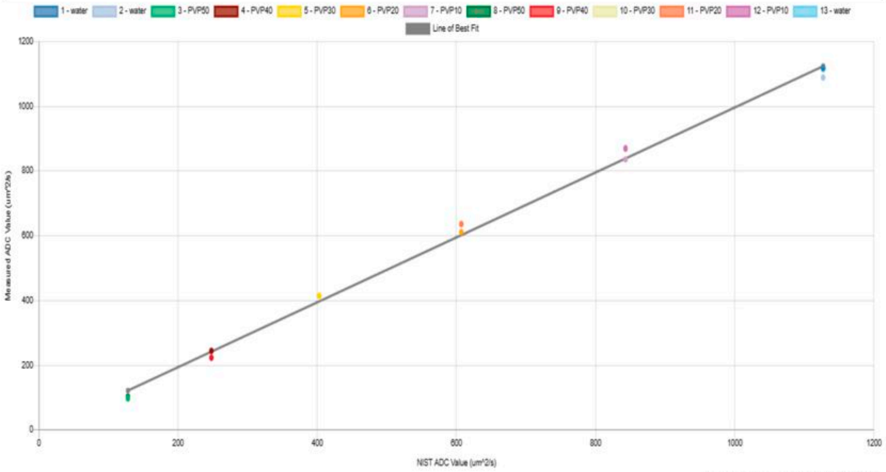

Best Fit Slope: 1.003 R^2: 0.997

NIST ADC Value vs wCV

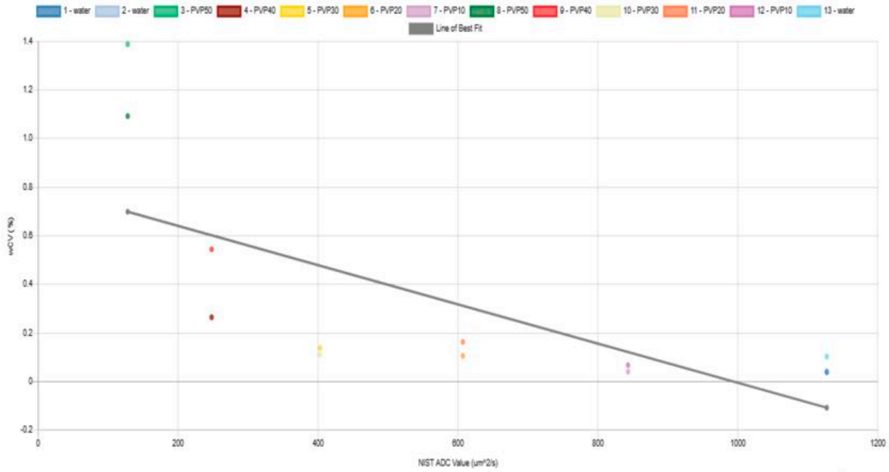

Best Fit Slope: -0.001 R^2: 0.492

ADC VOI Statistics

Label	Contents	# Voxels	NIST Value	Mean	Bias	Bias Percent	Max B-Value Dependence	SNR B0	SNR B1000	RC	Random Error	wCV
			um^2/s	um^2/s	um^2/s	um^2/s	% (associated b-value pair)			um^2/sec	%	%
Vial 1 - Limits					*abs() <= 40*	*abs() <= 3.6*	*< 2*	*>= 50*		*<= 15*	*<= 3.6*	*<= 0.5*
1	water	6340	1127	1110.2	-16.8	-1.5		380.3	215.8	2.91	0.43	0.09
2	water	6340	1127	1113.1	-13.9	-1.2		240.9	154.1	1.15	0.62	0.04
3	PVP50	6340	128	154.5	26.5	20.7		135.9	186.1	0.60	5.45	0.14
4	PVP40	6340	248	258.9	10.9	4.4		289.7	300.8	3.36	1.55	0.47
5	PVP30	6340	403	421.8	18.8	4.7		269.6	290.9	2.91	1.06	0.25
6	PVP20	6340	607	637.4	30.4	5.0		381.2	319.4	1.91	0.62	0.11
7	PVP10	6340	843	859.2	16.2	1.9		430.9	283.7	3.25	0.46	0.14
8	PVP50	6340	128	154.0	26.0	20.3		106.3	152.4	3.04	6.94	0.71
9	PVP40	6340	248	293.6	45.6	18.4		240.8	260.8	1.13	1.74	0.14
10	PVP30	6340	403	430.3	27.3	6.8		333.7	350.8	2.48	0.86	0.21
11	PVP20	6340	607	649.7	42.7	7.0		348.5	303.0	0.62	0.60	0.03
12	PVP10	6340	843	898.2	55.2	6.5		232.8	200.2	4.27	0.66	0.17
13	water	6340	1127	1123.3	-3.7	-0.3		332.9	199.2	3.73	0.44	0.12

ADC Value vs Axial Position

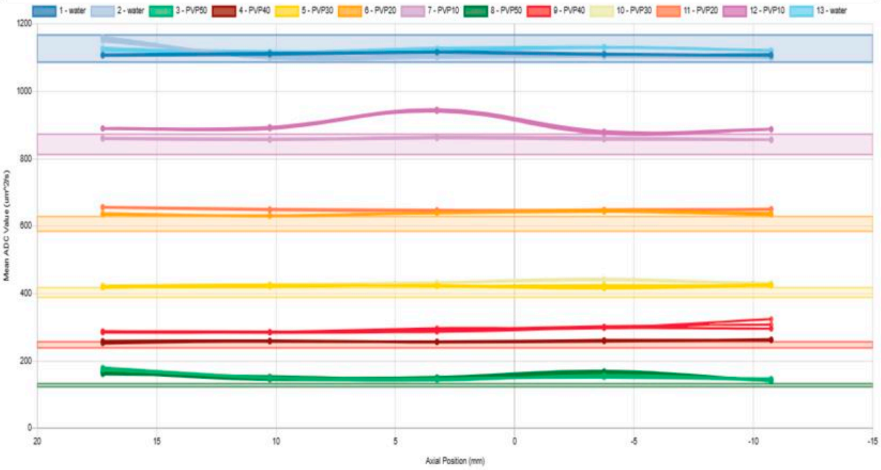

NIST ADC Value vs Measured ADC Value

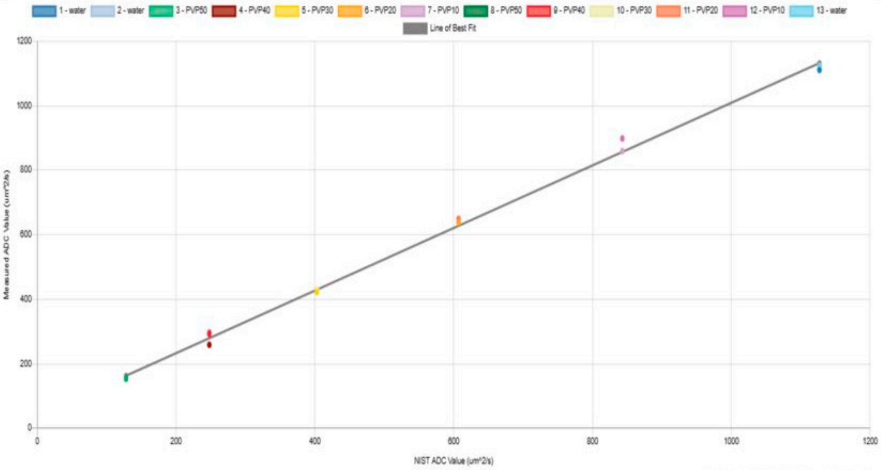

Best Fit Slope: 0.970 R^2: 0.998

NIST ADC Value vs wCV

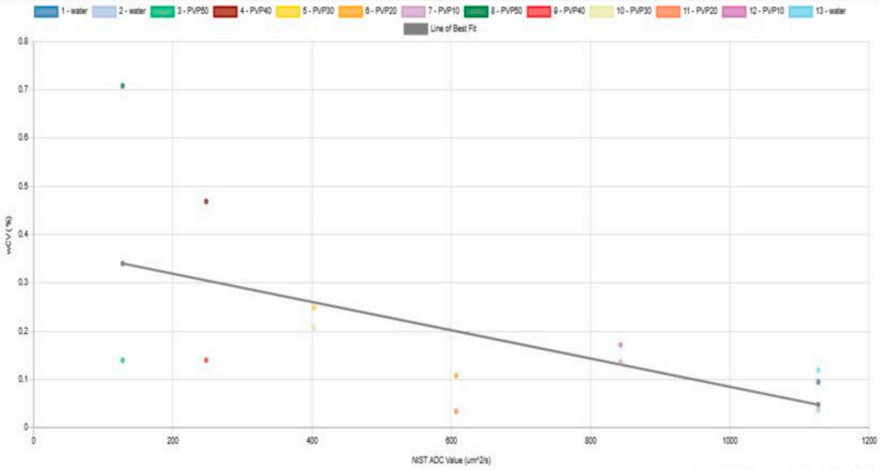

Best Fit Slope: -0.000 R^2: 0.347

ADC VOI Statistics

Label	Contents	# Voxels	NIST Value	Mean	Bias	Bias Percent	Max B-Value Dependence	SNR B0	SNR B1000	RC	Random Error	wCV
			um^2/s	um^2/s	um^2/s	um^2/s	% (associated b-value pair)			um^2/sec	%	%
Vial 1 - Limits					*abs() <= 40*	*abs() <= 3.6*	*< 2*	*>= 50*		*<= 15*	*<= 3.6*	*<= 0.5*
1	water	7608	1127	1118.7	-8.3	-0.7		199.7	141.4	3.14	0.79	0.10
2	water	7608	1127	1182.8	55.8	5.0		160.6	125.8	7.57	0.89	0.23
3	PVP50	7608	128	135.4	7.4	5.8		79.8	125.6	4.17	10.95	1.14
4	PVP40	7608	248	267.4	19.4	7.8		134.9	182.4	4.71	3.47	0.64
5	PVP30	7608	403	414.3	11.3	2.8		166.1	197.9	4.10	1.80	0.36
6	PVP20	7608	607	606.2	-0.8	-0.1		167.7	175.7	1.29	1.23	0.08
7	PVP10	7608	843	848.5	5.5	0.7		180.0	175.1	0.78	0.96	0.03
8	PVP50	7608	128	163.0	35.0	27.3		92.4	149.2	4.67	8.50	1.15
9	PVP40	7608	248	272.3	24.3	9.8		129.0	184.7	2.81	3.23	0.38
10	PVP30	7608	403	423.4	20.4	5.1		205.1	242.9	6.26	1.47	0.53
11	PVP20	7608	607	608.4	1.4	0.2		178.3	175.7	5.99	1.33	0.36
12	PVP10	7608	843	868.3	25.3	3.0		154.4	141.2	6.45	1.08	0.27
13	water	7608	1127	1137.0	10.0	0.9		205.9	159.0	4.16	0.67	0.13

ADC Value vs Axial Position

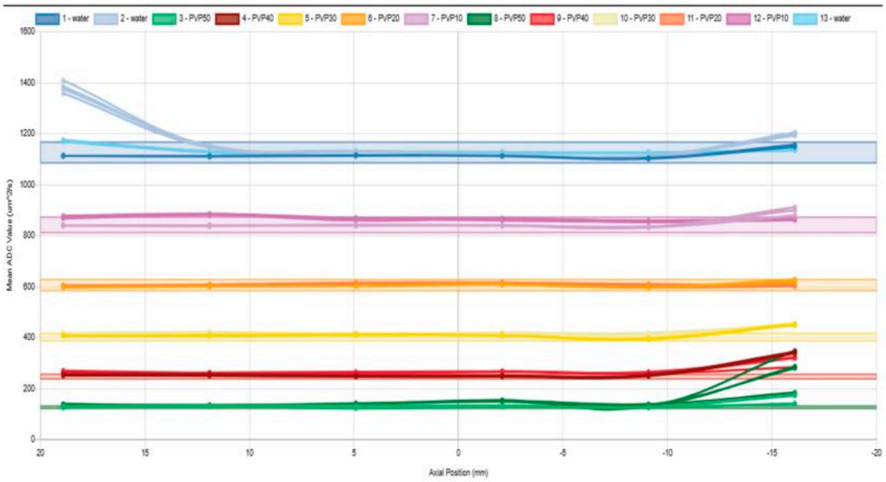

NIST ADC Value vs Measured ADC Value

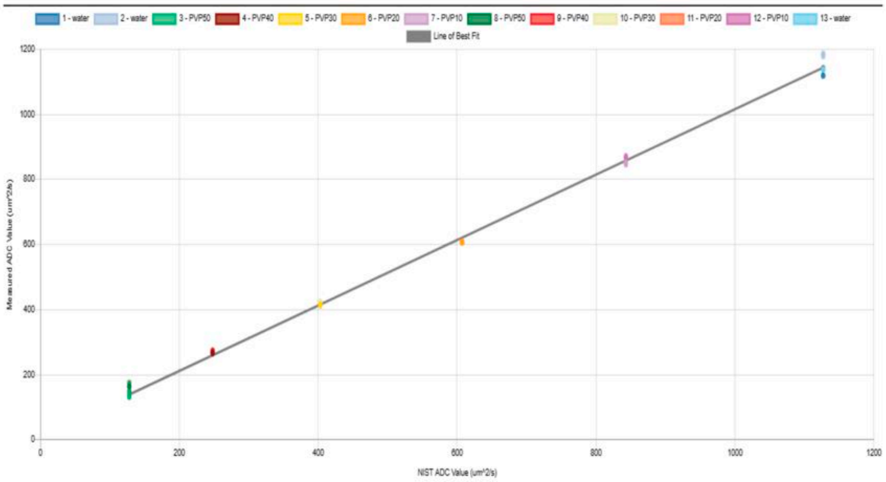

Best Fit Slope: 1.005 R^2: 0.998

NIST ADC Value vs wCV

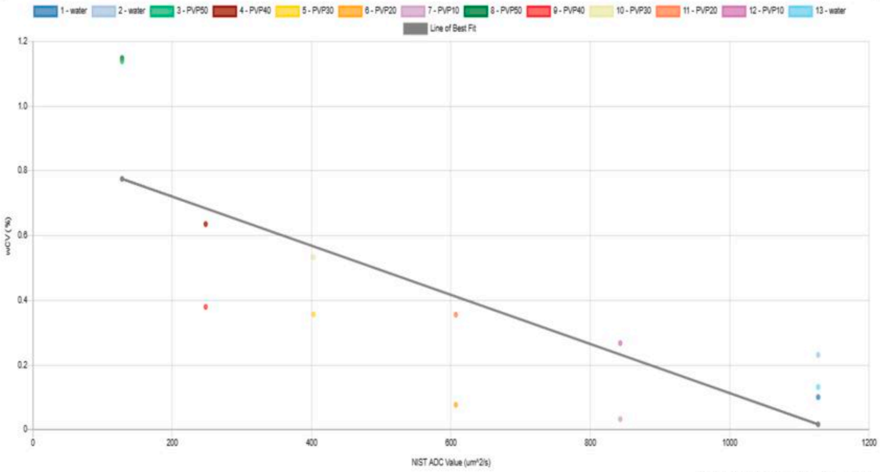

Best Fit Slope: -0.001 R^2: 0.610
